# Predicting bone metastasis-free survival in non-small cell lung cancer from preoperative CT via deep learning

**DOI:** 10.1038/s41698-024-00649-z

**Published:** 2024-07-28

**Authors:** Jia Guo, Jianguo Miao, Weikai Sun, Yanlei Li, Pei Nie, Wenjian Xu

**Affiliations:** 1 Department of Radiology, The Affiliated Hospital of Qingdao University, 266001 Qingdao, China; 2 College of Computer Science and Technology, Qingdao University, 266071 Qingdao, China; 3 Department of Radiology, Qilu Hospital of Shandong University, 250012 Jinan, Shandong China; 4 Third department of medical oncology, Qingdao Central Hospital, University of Health and Rehabilitation Sciences, Qingdao, China

**Keywords:** Cancer imaging, Outcomes research, Surgical oncology

## Abstract

Accurate prediction of bone metastasis-free survival (BMFS) after complete surgical resection in patients with non-small cell lung cancer (NSCLC) may facilitate appropriate follow-up planning. The aim of this study was to establish and validate a preoperative CT-based deep learning (DL) signature to predict BMFS in NSCLC patients. We performed a retrospective analysis of 1547 NSCLC patients who underwent complete surgical resection, followed by at least 36 months of monitoring at two hospitals. We constructed a DL signature from multiparametric CT images using 3D convolutional neural networks, and we integrated this signature with clinical-imaging factors to establish a deep learning clinical-imaging signature (DLCS). We evaluated performance using Harrell’s concordance index (C-index) and the time-dependent receiver operating characteristic. We also assessed the risk of bone metastasis (BM) in NSCLC patients at different clinical stages using DLCS. The DL signature successfully predicted BM, with C-indexes of 0.799 and 0.818 for the validation cohorts. DLCS outperformed the DL signature with corresponding C-indexes of 0.806 and 0.834. Ranges for area under the curve at 1, 2, and 3 years were 0.820–0.865 for internal and 0.860–0.884 for external validation cohorts. Furthermore, DLCS successfully stratified patients with different clinical stages of NSCLC as high- and low-risk groups for BM (*p* < 0.05). CT-based DL can predict BMFS in NSCLC patients undergoing complete surgical resection, and may assist in the assessment of BM risk for patients at different clinical stages.

## Introduction

Anatomical pneumonectomy is the primary treatment for early to mid-stage non-small cell lung cancer (NSCLC)^1^. Despite progress in endoscopic techniques and minimally invasive surgical treatments, recurrence rates remain elevated^2^, with approximately 15–40% of NSCLC patients experiencing bone metastasis (BM) during treatment^3,4^. The high risk of BM, which can induce skeletal-related events (SREs) such as pain, pathologic fracture, hypercalcemia, and spinal cord compression, has emerged as an important prognostic factor for NSCLC patients^5–8^.

For patients with suspected BM, emission computed tomography (ECT) or positron emission computed tomography (PET-CT) are routinely recommended^9–11^. However, the latest version of the National Comprehensive Cancer Network (NCCN) guidelines state that follow-up of stage I––IIIA asymptomatic NSCLC patients after localized treatment, routinely without ECT or whole-body PET-CT, is not timely in assessing the actual bone health status of patients with insidious BM. This limitation emphasizes the need for risk stratification of BM before performing postoperative follow-up^10^. Therefore, early screening of NSCLC with BM potential is important for guiding postoperative follow-up strategies, improving survival, and enhancing quality of life.

Deep learning (DL) techniques learn representative information from raw image automatically, and extract features in an incremental manner without human intervention. These characteristics enable decoding of relevant radiological tumor phenotypes, and provide additional information for planning subsequent treatment strategies^12–16^. Encouragingly, through the development of appropriate models with fine-grained features, DL has demonstrated promising potential across different applications to NSCLC such as lesion detection, lymph node metastasis prediction, and treatment response evaluation^17–20^. However, the training datasets used in previous studies did not consider important prognostic confounders, such as clinical T-stage, carcinoembryonic antigen (CEA) status and postoperative treatment^21,22^. As a consequence, the utility of the prognostic information extracted from CT image-based primary tumors remains uncertain. DL results must be integrated with known clinical prognostic factors to identify independent risk factors.

To address the above limitations, we established a DL-based multiparametric CT signature for predicting bone metastasis-free survival (BMFS) in NSCLC patients receiving surgery. For improved prediction, we also developed a deep learning clinical-imaging signature (DLCS) that combines imaging features with clinical variables, and we explored the utility of DLCS for assessing risk stratification of BM in NSCLC patients at different clinical stages.

## Results

### Baseline characteristics

Baseline patient characteristics are summarized in Table 1. Of 2832 resected NSCLC patients, we excluded 412 patients with distant metastasis at initial diagnosis, 341 patients with neoadjuvant radiochemotherapy, 354 patients without survival data, 52 patients with concurrent or heterochronous neoplasms, and 26 patients with lesions that were not measurable on CT images. We included 1547 NSCLC patients with clinical stage I–IIIA in this retrospective study. Sample sizes were 798, 470, and 279 for training, internal validation and external validation cohorts, respectively. The median age was 60 years (interquartile range (IQR): 54–67 years) for the training cohort, 61 years (IQR: 55–67 years) for the internal validation cohort, and 62 years (IQR: 55–68 years) for the external validation cohort. Of the 1547 patients, 296 (19%) experienced BM during an average follow-up period of 32 months (IQR: 25–46 months). Median BMFS times for the three different cohorts were 13, 16, and 12 months, respectively.

**Table 1:** Patient characteristics

Characteristic	Training cohort (*n* = 798)	Validation cohort		*P-*value
		Internal validation cohort (*n* = 470)	External validation cohort (*n* = 279)	
A. Clinical characteristics				
Age(year)^a^	60.4 ± 9.5	60.9 ± 9.6	61.1 ± 9.6	0.596
Gender				0.535
Men	421(52.8%)	252(53.6%)	158(56.6%)	
Women	377(47.2%)	218(46.4%)	121(46.3%)	
Smoking history				0.606
Never smoked	489(61.4%)	276(58.7%)	165(59.1%)	
Ex- or current smoker	308(38.6%)	194(41.3%)	114(40.9%)	
CEA(ng/mL)				0.097
<5	574(71.9%)	350(74.5%)	193(69.2%)	
5–20	144(18.0%)	84(74.5%)	47(16.8%)	
>20	80(10.0%)	36(7.7%)	39(14.0%)	
CYFRA21-1(ng/mL)	4.2 ± 11.1	4.1 ± 6.3	4.6 ± 7.6	<0.001
Pathologic type				0.101
Adenocarcinoma	606(75.9%)	331(70.4%)	217(77.8%)	
Squamous cell carcinoma	169(21.2%)	120(25.5%)	51(18.3%)	
Other	23(2.9%)	19(4.0%)	11(3. 9%)	
Clinical T category				0.010
cT1	484(60.7%)	297(63.2%)	140(50.2%)	
cT2	206(25.8%)	108(23.0%)	85(30.5%)	
cT3	70(8.8%)	49(10.4%)	40(14.3%)	
cT4	38(4.8%)	16(3.4%)	14(5.0%)	
Clinical N category				0.150
cN0	558(69.9%)	340(72.3%)	183(65.6%)	
cN1	240(30.1%)	130(27.7%)	96(34.4%)	
Postoperative treatment				0.305
None	540(67.7%)	322(68.5%)	173(62.0%)	
Adjuvant chemoradiotherapy	181(22.7%)	109(23.2%)	75(26.9%)	
Targeted therapy	28(3.5%)	28(6.0%)	8(2.9%)	
Combination therapy	49(6.1%)	11(2.3%)	23(8.2%)	
Bone metastasis (+)	147(18.4%)	94(20.0%)	55(19.7%)	0.759
B. Semantic features				
Nodule type				0.054
Nonsolid	131(16.4%)	87(18.5%)	52(18.6%)	
Part solid	103(12.9%)	43(9.1%)	20(7.2%)	
Solid	564(70.7%)	340(72.3%)	207(74.2%)	
Location				0.053
Central	177(22.2%)	94(20.0%)	43(15.4%)	
Peripheral	621(77.8%)	376(80.0%)	236(84.6%)	
Margin				0.246
Clear	458(57.4%)	284(60.4%)	175(62.7%)	
Unclear	340(42.6%)	186(39.6%)	104(37.3%)	
Presence of spiculation	245(30.7%)	139(29.6%)	103(36.9%)	0.089
Presence of lobulation	414(51.9%)	158(33.6%)	184(65.9%)	<0.001
Presence of air bronchogram	153(19.2%)	79(16.8%)	64(22.9%)	0.119
Presence of pleural attachment	288(36.1%)	156(33.2%)	74(26.5%)	0.014

Note: Unless otherwise noted, values are numbers of patients, with percentages in parentheses.

*P-*values were calculated by Kruskal-Wallis test, Chi-square test or Fisher exact test.

^a^Data are means ± standard deviations.

### DL signature predicts BM in the validation cohorts

**Fig. 1:**
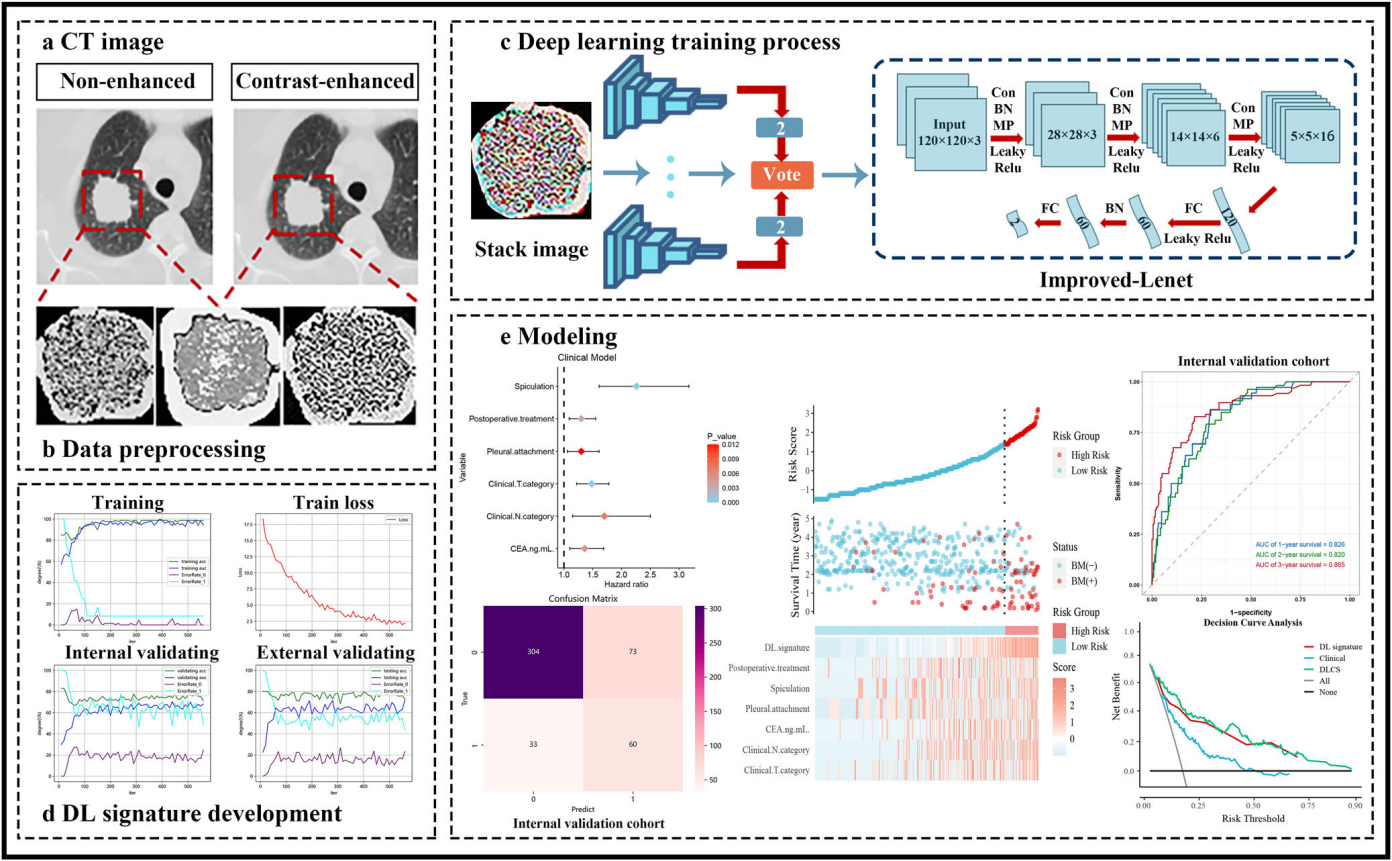
Flowchart of the study. Flowchart for developing the DL signature and DLCS for BM and prognosis prediction in NSCLC patients (*n* = 1547).

The training process of the deep CNN model based on multiparametric CT is shown in Fig. 1c, d, which shows that loss of the training cohort decreases and gradually converges with an increasing number of iterations. The DL-prob was generated by combining non-enhanced and enhanced CT images using the end-to-end DL method. The trained DL-prob successfully determines whether BM occurred within 3 years of surgery in NSCLC patients (Supplementary Fig. 1). DL-prob correlated closely with NSCLC patient prognosis, which demonstrates the feasibility of constructing reliable prognostic markers. Supplementary Fig. 2 shows the distribution of DL-prob predictions.

The DL signature developed using DL-prob demonstrated good prognostic predictive performance. When applied to the validation cohorts, the DL signature produced iAUC values of 0.825 and 0.848 for 3-year BMFS (Supplementary Fig. 3), and C-index values of 0.799 and 0.818 for BMFS (Table 2). The DL signature was found to provide valuable information for BM prediction, as shown by the attention maps (Supplementary Fig. 4). This analysis reveals areas associated with a high risk of suffering from BM through visualization of pixel weight distributions using different colors.

**Table 2:** The performance comparison of different models in validation cohorts

Cohorts	Model	C-index	95%CI	1 year-AUC	2 year-AUC	3 year-AUC
Internal validation	Clinical	0.715	0.664-0.767	0.757	0.753	0.772
	DL signature	0.799	0.751-0.847	0.766	0.768	0.825
	DLCS	0.806	0.766-0.846	0.826	0.820	0.865
External validation	Clinical	0.746	0.690-0.802	0.719	0.793	0.788
	DL signature	0.818	0.766-0.870	0.856	0.819	0.848
	DLCS	0.834	0.790-0.878	0.860	0.864	0.884

*DL* deep learning, *DLCS* deep learning radio-clinical signature, *CI* confidence interval.

### DLCS construction and prognostic performance evaluation

We performed univariate and multivariable Cox analyses to determine the association between important clinical-imaging variables and BMFS. These analyses revealed that Clinical T category, Clinical N category, CEA, postoperative treatment, pleural attachment, and spiculation were prominently associated with BMFS, resulting in the development of the clinical model (Table 3). We combined the DL signature with six important clinical-imaging variables to establish the DLCS. SHAP analysis elucidated that each variable effectively contributed to the model’s decision-making process (Supplementary Fig. 5).

**Table 3:** Univariable and multivariable analyses of bone metastasis-free survival in patients with resected non-small cell lung cancer

	Univariate			Multivariate		
Variable	HR	95%CI	*P*-value	HR	95%CI	*P*-value
Gender	0.74	0.54-1.04	0.08			
Age	1.01	0.99-1.03	0.372			
Clinical T category	1.91	1.64-2.22	<0.001^*^	1.38	1.11-1.70	0.003^*^
Clinical N category	3.74	2.69-5.2	<0.001^*^	1.51	1.01-2.26	0.044^*^
Smoking	1.37	0.99-1.89	0.06			
Pathologic type	1.36	1.02-1.82	0.035^*^	1.17	0.77-1.77	0.460
CEA (ng/mL)	2.17	1.79-2.63	<0.001^*^	1.31	1.05-1.64	0.016^*^
CYFRA21-1(ng/mL)	1.01	1.00-1.01	0.001^*^	1.00	1.00-1.01	0.187
Location	1.80	1.27-2.55	0.001^*^	1.50	0.98-2.29	0.064
Nodule type	0.41	0.29-0.58	<0.001^*^	0.88	0.59-1.31	0.524
Margin	1.61	1.17-2.23	0.004^*^	1.41	0.91-2.19	0.126
Lobulation	1.79	1.28-2.51	0.001^*^	1.01	0.66-1.53	0.966
Spiculation	2.42	1.75-3.34	<0.001^*^	2.20	1.52-3.20	<0.001^*^
Air bronchogram	1.33	1.14-1.55	<0.001^*^	1.00	0.84-1.19	0.968
Pleural attachment	1.68	1.36-2.07	<0.001^*^	1.29	1.04-1.59	0.021^*^
Postoperative treatment	1.83	1.59-2.11	<0.001^*^	1.33	1.11-1.59	0.002^*^

*HR* hazard ratio, *CI* confidence interval.

*P-value less than 0.05.

**Fig. 2:**
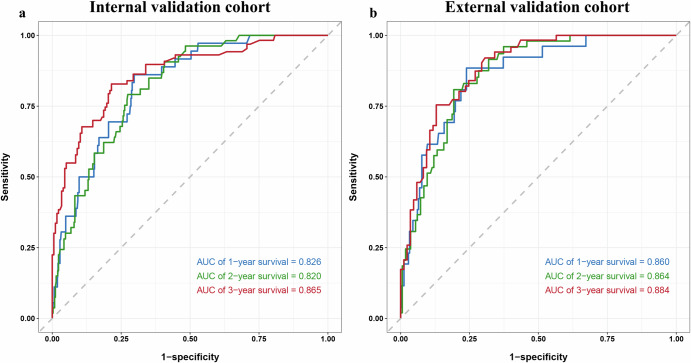
Predictive efficacy of DLCS on BMFS. (**a**) and (**b**) represent time-dependent ROC curves for the internal (*n* = 470) and the external (*n* = 279) validation cohorts.

**Fig. 3:**
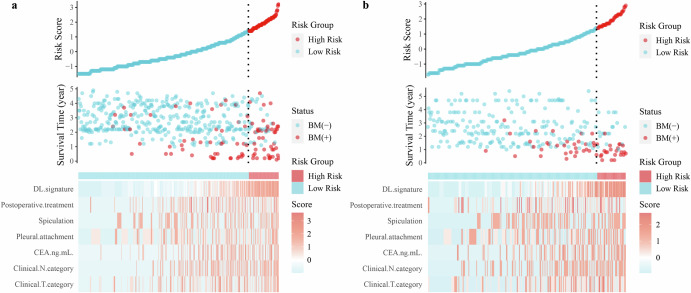
DLCS risk score for BMFS. (**a**) and (**b**) are risk stratification and distribution of clinical-imaging characteristics of high- and low-risk patients with BM among internal and external validation cohorts, respectively.

**Fig. 4:**
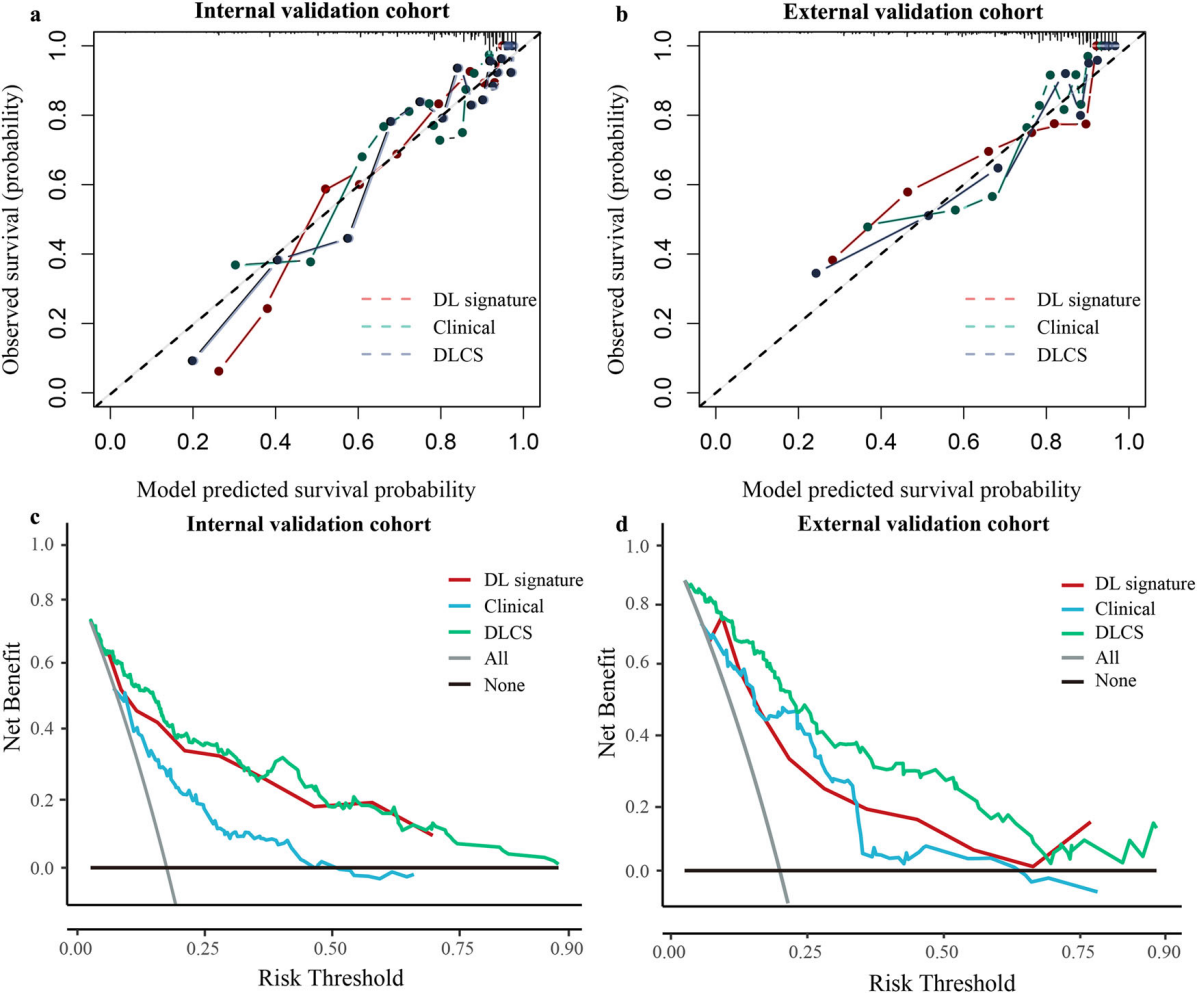
Evaluation of DLCS in multicentre cohorts. Figure (**a**, **b**) and (**c**, **d**) shows the calibration and decision curves for the three models in the internal (*n* = 470) and external (*n* = 279) validation cohorts, respectively.

Compared with other competing models, DLCS exhibited significantly better performance (Table 2). DLCS produced the highest C-index for both internal and external validation cohorts with values of 0.806 (95% CI: 0.766–0.846) and 0.834 (95% CI: 0.790–0.878), respectively. When using internal and external validation cohorts, the risk scores generated by DLCS accurately predicted BMFS at 1, 2, and 3 years (Fig. 2). Based on DLCS outputs, patients were divided into high- and low-risk groups for BM. The optimal critical value of the DLCS for the training cohort was determined to be 1.39. Figure 3 illustrates the compositional feature distribution based on high and low DLCS scores. Calibration curves for three models showed good agreement between predictions and observations across validation cohorts (Fig. 4a, b). Using net benefits as the evaluation indicator for the decision curve, DLCS presents excellent benefits within the relevant thresholds (Fig. 4c, d).

### BM risk stratification based on DLCS

**Fig. 5:**
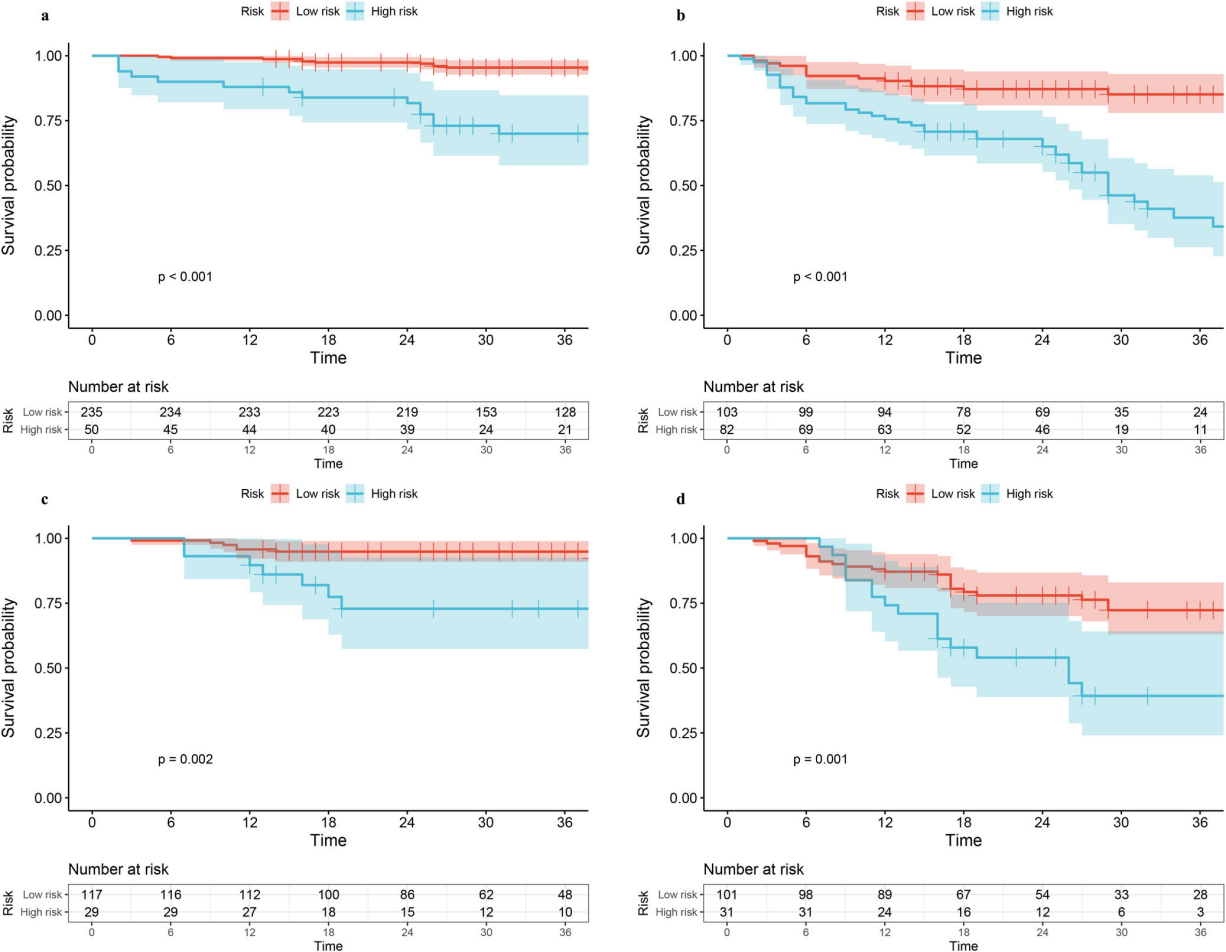
DLCS-based KM curves of patients at stage I and II-IIIA. Figure (**a**, **b**) and (**c**, **d**) shows the KM BMFS curves for the subgroups of stage I and stage II-IIIA patients in the internal and external validation cohorts, respectively. At the bottom of the graph is the distribution of patients at different risk for each time point.

We performed subgroup BM risk analysis based on DLCS by classifying patients into high- and low-risk groups. Figure 5a–d demonstrates that BMFS values of patients at stage I and II–IIIA within both internal and external validation cohorts were significantly stratified by risk score (*p* < 0.05, log-rank test).

## Discussion

Accurate prognostic prediction is useful for selecting therapeutic strategies and stratifying patient risk. In this study, we established and validated a DL-based multiparametric CT signature for predicting BMFS in NSCLC patients undergoing surgical treatment. The DL signature proved to be reliable for BM prediction and risk stratification, demonstrating its value in prognostic prediction. The DLCS with multidimensional information integrating the DL signature and BM-associated clinical imaging features performed better in predicting BMFS.

Patients with NSCLC accompanied by BM usually present an unfavorable prognosis, significantly reducing their quality of life. Follow-up strategies in NSCLC patients are routinely based on clinical practice guidelines, including guidelines from the NCCN^10^ and the European Society for Medical Oncology (ESMO)^23^. For patients with surgically available clinical stage I–IIIA NSCLC, the same follow-up management protocol was recommended. If all patients were to undergo skeletal examination only after SREs appeared, BMFS would not be guaranteed in patients with unfavorable prognosis, while disease progression in patients with asymptomatic BM would be overlooked. In this study, the established DLCS can be used as an effective prognostic tool for classifying patients as high and low risk for BM regardless of disease stage, which may contribute to more useful personalized follow-up strategies. The percentages of patients within the stage I cohort considered at high risk for BM according to the DLCS were 16.1% (79/490, training cohort), 17.5% (50/285, internal validation cohort), and 13.0% (29/146, external validation cohort). In the stage II–IIIA cohort, the DLCS classified 28.9% (89/308) of patients as high-risk for BM in the training cohort, 28.6% (53/185) in the internal validation cohort, and 23.3% (31/133) in the external validation cohort. The above results indicate that tumor load and individualized BM risk may vary significantly among patients, even when corresponding to the same clinical stage. Postoperative skeletal examination should not be omitted in high-risk patients, as early detection of BM lesions is crucial for aggressive salvage therapy. In future clinical applications, if the DLCS defines patients at clinical stages I–IIIA as being at high risk of BM, bone-related screening should be performed carefully in the follow-up strategy, rather than relying on patient requests for examination following manifestation of symptoms.

The “watch-and-wait” strategy is currently safe and effective in postoperative NSCLC patients^23^. Because advances in therapeutic techniques have improved the overall survival of patients with lung cancer, the importance of bone invasion has become increasingly significant. Proactive prevention of BM in high-risk individuals is associated with significantly better outcomes than reactive treatment approaches^24^. This observation emphasizes the importance of early identification of those at risk, so that appropriate interventions can be implemented promptly. Bone-targeting agents that complement cancer-specific therapies, such as denosumab and bisphosphonates, have been proven to reduce the risk of SREs by improving bone structure and quality^11,25^. The risk scores calculated by our DLCS model can help identify high-risk patients who may benefit from more aggressive treatment interventions, even at an advanced clinical stage. This valuable contribution can be used to develop strategies for improving the decision-making process associated with the watch-and-wait approach, by providing objective data-driven insights into individual patient risks.

This study extracted information from multiparametric CT scans using a DL method. Findings from previous studies have demonstrated that machine-learning techniques can extract useful characteristics from CT scans to forecast distant metastasis in NSCLC patients. These techniques can be used to investigate the correlation between clinical-semantic features and distant metastases, aiding in assessing prognostic outcomes^26–28^. Radiomic techniques successfully capture peri-tumoral and intra-tumoral features associated with image voxels, revealing tumor heterogeneity phenotypes and providing prognostic information. These methods present some limitations as they rely on predetermined representations set by domain experts, potentially leading to the omission of image features that are relevant to a particular task. The process of cancer distant metastasis follows a recognizable pattern and occurs selectively rather than randomly^29,30^. Metastastic processes selective for specific organs are collectively known as “organotropic metastasis” in the literature^31,32^. Neoplasms are genetically and histopathologically heterogeneous, manifesting as spatially variable within the tumor. Tumors with a high degree of heterogeneity display different metastatic propensities as a consequence of their intrinsically aggressive biology. Based on the above considerations, we proposed the end-to-end DL-prob for BM assessment in completely surgically resected NSCLC patients. This approach integrates non-enhanced and enhanced CT images to extract more comprehensive tumor information from multicentric datasets for classification purposes. More specifically, we employed an intermediate fusion strategy to select interpretable enhancement methods for each modality, and to address feature imbalance, lost modalities, and coordinated representation learning while capturing the underlying biological relationships between modalities^33^. Different network types are applied to each modality to narrow the heterogeneity gap and enable effective imaging fusion^34^. We then established a DL signature based on the time to postoperative BM, which proved to be highly correlated with prognosis. The DL signature carries great methodological potential for capturing multi-parameter imaging information and for making prognostic predictions, as it captures relevant information specific to the task. In order to determine whether the model may produce a positive or a negative impact on the field of image-based CNN, we generated activation maps to capture high-weighted image regions associated with network prediction. These maps make it possible to visualize activation regions with high-risk trends. We hypothesized that the areas highlighted by the activation maps may be associated with tumor progression, thus providing additional confidence in the predictive ability of the DL signature. This approach enables objective identification of high-risk areas for BM in tumor, which is invaluable for clinical risk assessment and precise clinical intensification of treatment regimens.

There is consensus that prognosis in cancer patient is closely connected with certain clinical features. TN staging and postoperative adjuvant therapy correlate with prognosis in NCSLC patients, in agreement with our findings^35,36^. The cell adhesion-associated glycoprotein CEA is widely regarded as a marker for tumor invasiveness, with increased CEA levels potentially promoting tumor cell infiltration and metastasis. Multifactorial Cox regression analysis showed that serum CEA levels (>5 µg/L) were directly related to the occurrence of BM. We also considered potential CT semantic features. Previous research indicates that pleural attachment and burr signs serve as imaging markers for tumor aggressiveness^37,38^. Within clinical models, pleural attachment and burr signs are independent risk predictors of BM, presumably owing to interstitial changes in tumor tissue growth and exudation along vascular, bronchial, or lobular septa raising the risk of BM occurrence. Several studies have shown that the integration of clinical-imaging information into machine learning models can enhance their prognostic prediction^39,40^. We confirmed these prior findings by combining several prognostically relevant clinical imaging factors with multiparametric deep CT features to develop a multidimensional prognostic model with superior performance.

Our study presents several limitations. First, we focused on the entire population of NSCLC patients. Different pathological types of NSCLC present various radiological characteristics and tumor burden, which may lead to heterogeneity in BM risk and prognosis. As a result, the validity of our model for BM assessment in different histological subtypes needs to be further confirmed by future studies using larger cohorts. Second, the DL signature is not fully automated, requiring manual marking of the tumor on CT images. Third, we collected external validation from healthcare organizations with a potentially similar distribution of patient characteristics, making it necessary to perform further international validation on a larger scale before applying the model to other races. Finally, the main limitation of DL for medical image analysis is its lack of interpretability that inevitably arises from its black box nature. Although we are able to visualize DL features extracted from tumors, their explicit biological meaning remains unknown and will require further clarification. Future studies will need to unravel the opacity of DL networks.

In conclusion, we proposed a DL-based multiparametric CT model to predict BM in surgically resectable NSCLC patients, and we constructed a DLCS by combining deep multiparametric CT information with clinical imaging factors to further improve their clinical application. Both DL signature and DLCS showed good performance in predicting BM. The output generated by DLCS can serve as a useful tool for BM risk stratification of different clinical stages of NSCLC, potentially assisting clinical decisions in precision medicine.

## Methods

### Ethics

This study complied with the Declaration of Helsinki and was approved by the Medical Ethics Review Board of Affiliated Hospital of Qingdao University. Because this study involved retrospective data analysed anonymously, written informed consent was waived. Figure 1 summarizes the design of our study.

### Patients

We retrospectively collected CT images and clinical information from patients with clinical stage I-IIIA NSCLC who underwent complete surgical resection between January 2018 and December 2019. We used 798 patients from the Affiliated Hospital of Qingdao University to construct the training cohort, 470 patients from the Affiliated Hospital of Qingdao University to construct the internal validation cohort, and 279 patients from Qilu Hospital of Shandong University to construct the external validation cohort. We included patients with NSCLC receiving complete surgical resection. We adopted the following exclusion criteria: preoperative neoadjuvant radiochemotherapy; more than 2 weeks between preoperative CT examination and operation; initial diagnosis of distant metastasis; concurrent or heterochronous tumor; non-measurable lesions on CT images; absence of clinical or survival data.

### Data collection

Initial clinicopathological information for each patient was extracted from the medical record system, including age, sex, history of smoking, histological subtype, CEA status, CYFRA21-1 and postoperative treatment. We determined clinical T-stage and N-stage in accordance with the American Joint Committee on Cancer (AJCC) 8th Edition Lung Cancer Staging System. Two radiologists with 6 and 3 years of clinical experience, who were blinded to clinicopathological information, independently evaluated the following CT semantic features: location, nodule type, margin, spiculation, lobulation, air bronchogram, and pleural attachment. In the event of disagreement, discussions were carried out to reach consensus. The endpoint event was the presence or absence of BM and documentation of BMFS, defining the interval between surgery and the date of first confirmation of BM (confirmed by imaging and histological evidence). Follow-up endpoints were obtained from outpatient medical records and telephone interviews.

### CT imaging acquisition

The patients in the training and internal validation cohorts underwent preoperative chest CT scans using five different scanners from four vendors [Brilliance 128, Philips Healthcare, Andover, MA; Revolution 256, and Optima 670, GE Medical Systems, Milwaukee, WI; Definition 129, Siemens Healthcare, Erlangen, Germany; Aquilion One TSX301A, Toshiba Medical Systems, Otawara, Japan]. The CT images in the external validation cohort were taken with three scanners from two manufacturers [Optima 620 and Optima 660, GE Medical Systems, Milwaukee, WI; Perspective 64, Siemens Healthcare, Erlangen, Germany]. Heterogeneity in the imaging acquisition protocols was inevitable as data were obtained retrospectively with different scanners.

All patients received CT scans from the apex to the bottom of the lungs while suspended for maximum inhalation. Scans were performed at 120 kVp with mAs ranging at 20–200 mAs with or without automatic exposure control according to the capability of each scanner. CT scan reconstruction of the slice thickness is less than or equal to 5 mm. Slice increments were equal to or less than slice thickness. All CT scans included axial reconstruction. All patients received enhanced CT scans after contrast material injection with a delay of 60-70 s.

### Data preprocessing

For both non-enhanced and enhanced CT images, we identified the region containing the largest cross-sectional area of the entire tumor, followed by image normalization and standardization. To focus on the tumor while exploring the surrounding tissue, we set the pixel values outside the region of interest (ROI) to 0 and used image erosion techniques to expand the ROI for cropping. This strategic approach allows us to preserve essential pixels around the tumor, ensuring comprehensive consideration of lesion information. The cropped image was converted to a greyscale single-channel image. Non-enhanced images were sharpened (sharpening intensity = 5) using a Laplace operator for better extraction of edge features. The risk probability of BM was generated by combining non-enhanced CT images, enhanced CT images, and sharpened images into three-channel images, which were used as input to the DL model. For image size uniformity, we resized all input images to 120 × 120 pixels using linear interpolation.

### DL signature development

The DL signature was developed using a 3D convolutional neural network (CNN) operating on the training cohort. The network contains a total of four convolutional layers (kernel sizes of 3 × 3 × 12, 3 × 6 × 5, 6 × 16 × 5, 16 × 120 × 5). The first three convolutional layers are followed by the application of a BN layer, a LeakyRelu activation function, and a pooling layer. Two fully connected layers are also included, and the BN layer is inserted between the two fully connected layers. Different enrolment groups were formed depending on whether BM occurred during the 3 postoperative years, and the DL model in the input graph bounding box was trained to obtain the probability of each patient developing BM within 3 years of surgery. This probability denotes the metastatic probability and the non-metastatic probability (summing to 1), and is defined as the DL-prob. The DL-prob was then used to construct the DL signature using the Cox proportional hazard model.

Throughout the training process, the Adam algorithm was used to train the neural network at an initial learning rate of 1 e-5. We applied a binary cross-entropy function to stabilize loss at 0.1. Code implementation relied on PyTorch. The algorithm was trained on a computer with an Intel i7-13700HX 16-core CPU and an RTX 4080 12GB GPU. We performed combined prediction of multiple trained models using the method of ensemble learning with hard voting. To reduce generalization error and increase model diversity, we randomly selected 80% of the training cohort data each time when training a model. After training multiple models, calculating the average predicted class of these models helps prevent severe impact from individual model prediction errors on the entire ensemble model, thereby achieving stronger robustness. To visualize image regions that are important for prediction, we generated attention maps using gradient-weighted class activation mapping to detect identified and monitored lesion regions.

### Individualized DLCS construction and model performance evaluation

Combining clinical-imaging features with multiparametric CT deep features can further enhance model performance. We applied Cox risk regression analysis to the training cohort to screen for independent predictors and construct the clinical model. We then constructed the DLCS by combining the DL signature with the clinical model, and converted the DLCS into an individualized BM prediction nomogram.

### DLCS-based BM risk assessment in patients at different clinical stages

Different clinical stages call for different treatment strategies 10. NSCLC patients were categorized into two subgroups according to AJCC staging: stage I and stage II–IIIA. We investigated whether DLCS could stratify patients associated with different BM risk levels in these cohorts using subgroup analysis. Specifically, we used the DLCS threshold of the Youden index to stratify patients into BM high-risk and low-risk. We then assessed postoperative BMFS for patients in different risk groups using Kaplan-Meier (KM) survival analysis.

### Statistical analysis

All statistical analyses were implemented using R software (version 4.0) and SPSS (IBM, version 22.0). We used Student’s *t* test, ANOVA, or Kruskal-Wallis tests to analyze continuous variables. We used chi-square tests or Fisher’s exact tests to compare categorical variables. We implemented receiver operating characteristic (ROC) analysis for the BM classification model using continuous probability scores ranging between 0 and 1. We calculated the integrated area under the time-varying ROC curve (iAUC) for survival models, and evaluated discriminatory performance using Harrell’s concordance index (C-index). We evaluated the clinical practicality of the BM prediction model using decision curve analysis, which quantifies the net benefit at different threshold probabilities. We relied on calibration curves to evaluate consistency of the predicted probabilities with actual observations. We used Cox regression to assess prognostic elements and analyze the multivariable-adjusted hazard ratio. Additionally, we performed KM survival curve analyses based on BMFS to validate the discrimination ability of the model using the log-rank test. We defined statistical significance as *p* < 0.05.

### Supplementary information

Supplementary Files

## Data Availability

The data can be obtained from the corresponding author upon reasonable request approved by the Institutional Review Board of all registered centers.

## Code availability

The underlying code for this study is available in github and can be accessed via this link (https://github.com/DRGUOJ/NSCLC-BM).
