# Supplementary material for: Predicting bone metastasis-free survival in non-small cell lung cancer from preoperative CT via deep learning
Source: NPJ Precis Oncol. 2024 Jul 28;8:161. doi: 10.1038/s41698-024-00649-z (PMC11283482; doi:10.1038/s41698-024-00649-z)
Supplement: Supplementary file 1 — Supplementary Files [file 41698_2024_649_MOESM1_ESM.pdf]

Supplementary material

Figure

Supplementary Figure 1

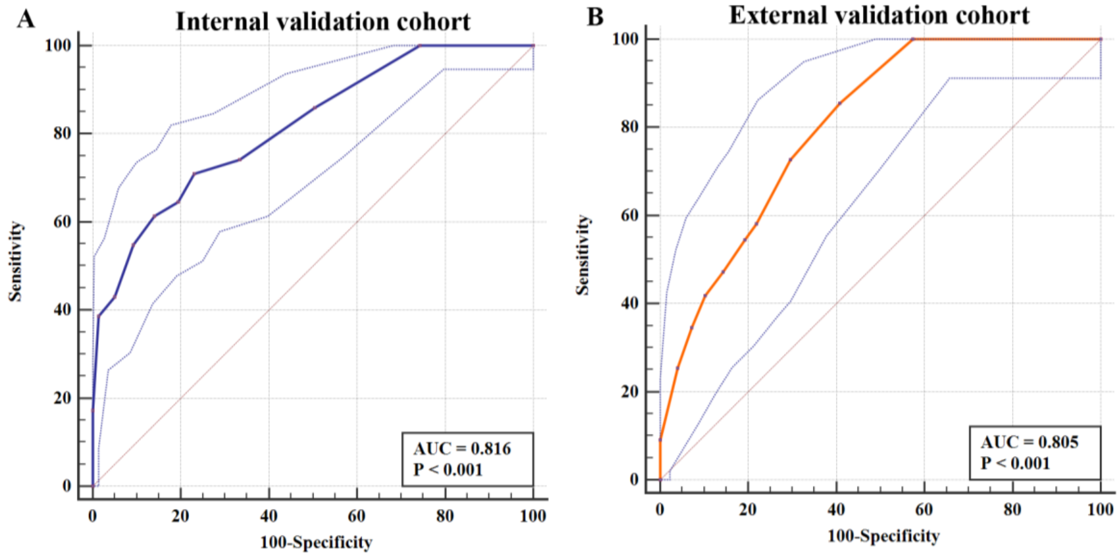

ROC curves of prognostic performance with DL-prob of the (A) internal validation cohort (n = 470) and (B) external validation cohort (n = 279). DL: deep learning; ROC: receiver operating characteristic; AUC: area under receiver operating characteristic curve.

Supplementary Figure 2

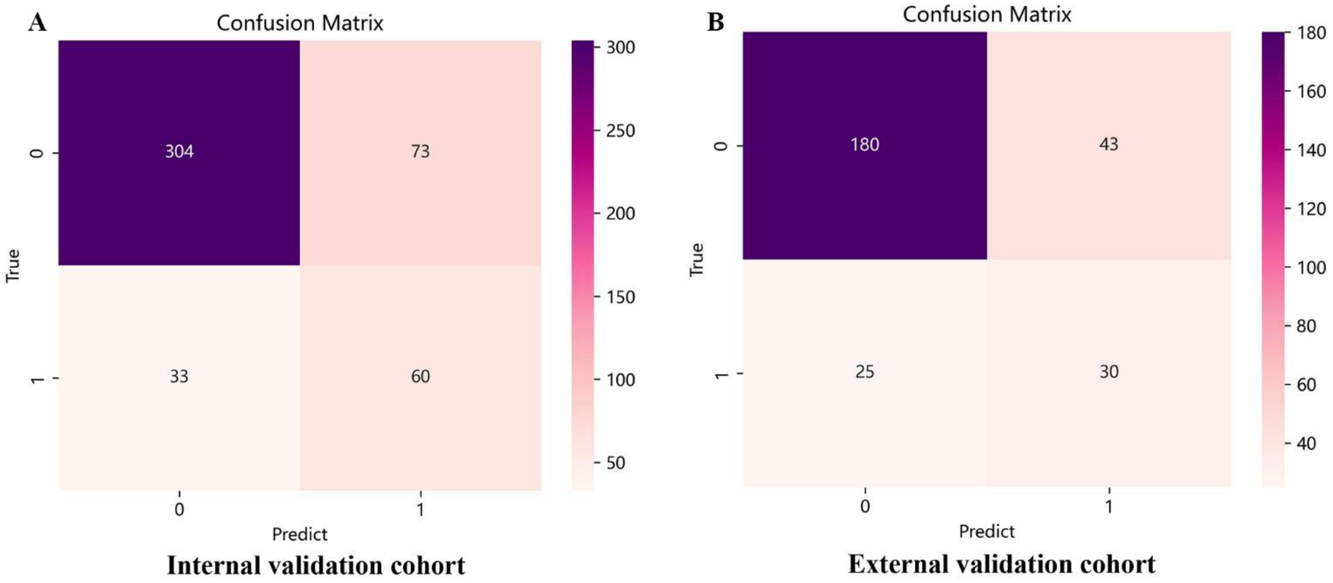

Distribution of DL-prob predictions of the (A) internal validation cohort (n = 470) and (B) external validation cohort (n = 279). DL: deep learning.

Supplementary Figure 3

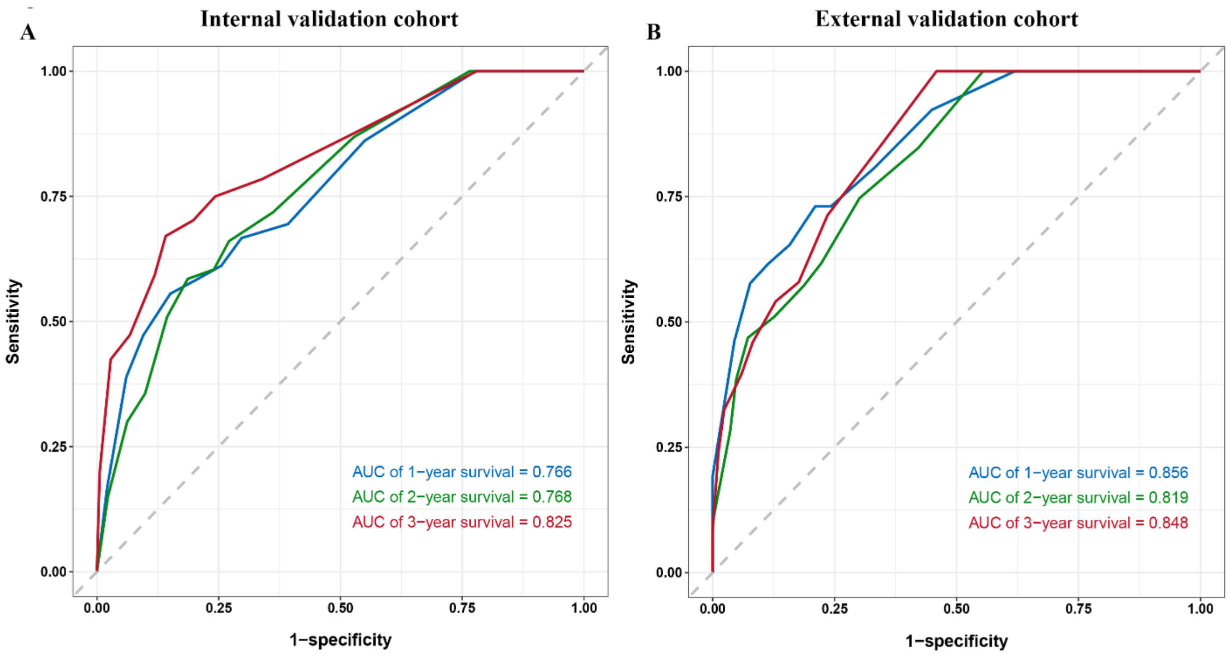

Predictive performance of DL signature for BMFS. (A) and (B) are time-dependent ROC curves for one year, two years and three years of the internal validation cohort (n = 470) and external validation cohort (n = 279). DL: deep learning; BMFS: bone metastasis-free survival; ROC: receiver operating characteristic.

Supplementary Figure 4

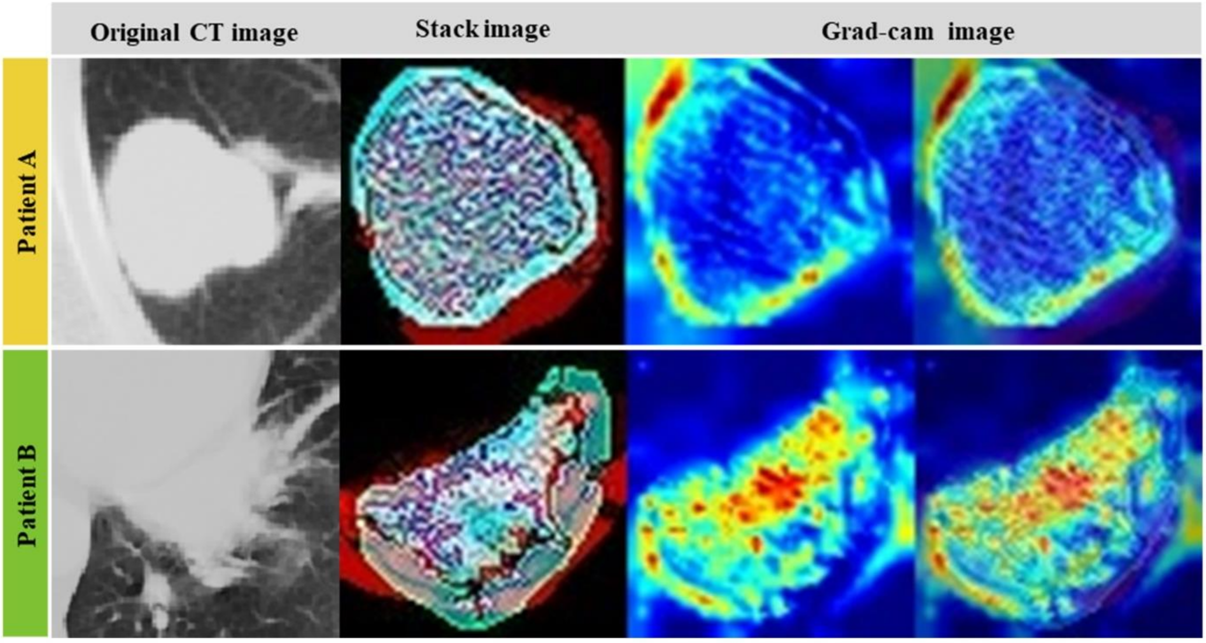

Representative CT images overlaid with attention maps for the model interpretation. Patient A was a 55-year-old man with clinical stage 3a NSCLC who did not develop bone metastasis during the follow-up period after resection. No significant highlighted areas are seen in the activation map. Patient B was a 72-year-old man with clinical stage 2b NSCLC that bone metastasis 20months after resection. Tumor and its peritumoral rim are activated by the CT-based deep learning prediction model.

Supplementary Figure 5

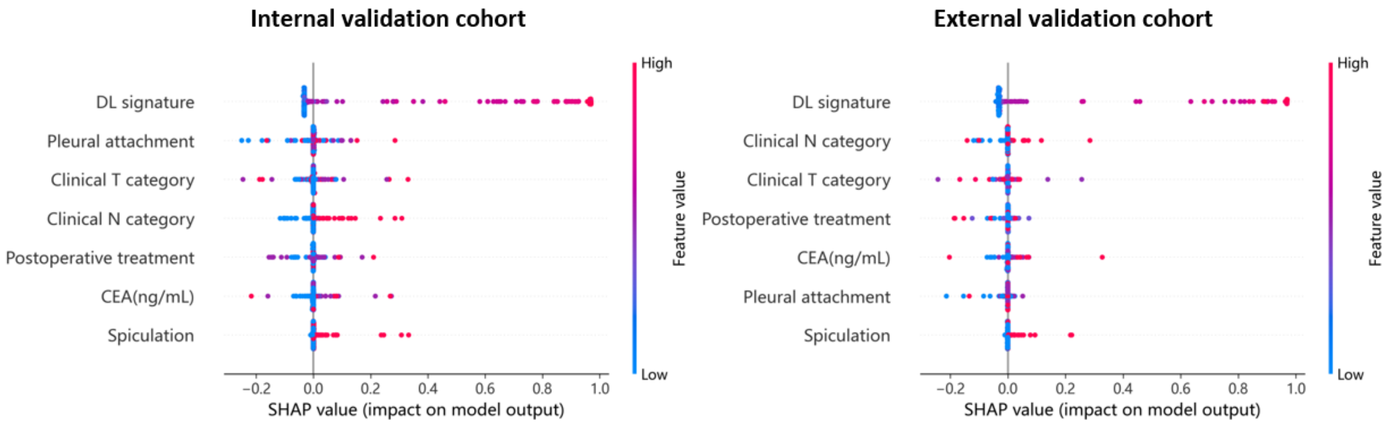

SHAP summary dot plot. The probability of BM development increases with the SHAP value of a feature. A dot is made for SHAP value in the model for each single patient, so each patient has one dot on the line for each feature. The colors of the dots demonstrate the actual values of the features for each patient, as red means a higher feature value and blue means a lower feature value.
